# The Many Faces of Meckel’s Diverticulum: Clinical Presentations and Complications

**DOI:** 10.7759/cureus.90370

**Published:** 2025-08-18

**Authors:** Shishir Kumar, Mrunalkant Panchal, Pranay Mishra, Preeti Srivastava, Sanjay K Tanti

**Affiliations:** 1 Department of Surgery, Tata Main Hospital, Jamshedpur, IND; 2 Department of Surgery, Manipal Tata Medical College, Manipal Academy of Higher Education, Jamshedpur, IND; 3 Department of General Surgery, Tata Main Hospital, Jamshedpur, IND; 4 Department of Pediatrics, Manipal Tata Medical College, Manipal Academy of Higher Education, Jamshedpur, IND; 5 Department of Pediatrics, Tata Main Hospital, Jamshedpur, IND

**Keywords:** ectopic gastric mucosa, gastrointestinal bleeding, ileostomy, intestinal obstruction, meckel’s diverticulum

## Abstract

Background

Meckel’s diverticulum (MD) is a congenital anomaly of the gastrointestinal tract characterized as a true diverticulum that contains all three layers of the intestinal wall. It is typically situated on the antimesenteric border of the terminal ileum and receives blood supply from the vitelline artery. Although often asymptomatic, MD can lead to complications such as intestinal obstruction, gastrointestinal bleeding, perforation with peritonitis, and, rarely, malignancy. Histologically, ectopic gastric and pancreatic tissues are the most commonly observed findings.

Methods

This retrospective study reviewed pediatric cases of symptomatic MD treated between June 2022 and May 2025. Data regarding clinical presentation, operative procedures, histopathological findings, and treatment outcomes were analyzed.

Results

The average age at presentation was 40 months, ranging from one to 60 months. Among the six children studied, two (33%) presented with umbilical discharge, and two (33%) had intestinal obstruction, one (16%) of whom had associated bowel gangrene. These four (64%) patients underwent laparotomy with ileal resection and anastomosis. One (16%) child presented with perforation peritonitis due to a ruptured MD and required an emergency ileostomy, later reversed after eight months. One (16%) child, who had intermittent lower gastrointestinal bleeding and anemia, underwent laparoscopy-assisted Meckel’s diverticulectomy. Histopathology revealed ectopic gastric mucosa in five (84%) patients and pancreatic tissue in one (16%) patient; no malignancy was identified.

Conclusion

Prompt surgical resection with restoration of bowel continuity is crucial in cases of symptomatic MD. Although resection of incidentally discovered MD during unrelated abdominal procedures is sometimes considered due to its potential for future complications, including bleeding or malignancy, there is currently insufficient evidence to support the routine removal of asymptomatic MD in the absence of other abdominal pathology.

## Introduction

Meckel’s diverticulum (MD) is the most common congenital anomaly of the gastrointestinal tract, resulting from the incomplete obliteration of the vitelline (omphalomesenteric) duct during embryonic development. It is classified as a true diverticulum because it comprises all three layers of the intestinal wall, i.e., mucosa, submucosa, and muscularis propria, unlike false diverticula, which lack muscular layers [1]. Anatomically, MD is located on the antimesenteric border of the terminal ileum, typically within 60 cm (2 feet) of the ileocecal valve, and derives its vascular supply from the persistent vitelline or omphalomesenteric artery [2].

A widely accepted clinical heuristic, the “rule of twos,” is often used to characterize MD: it occurs in approximately 2% of the general population, is twice as common in males as in females (2:1), is found within 2 feet of the ileocecal valve, measures roughly 2 inches in length and 2 cm in diameter, may contain two types of heterotopic tissue (commonly gastric and pancreatic), and is often diagnosed before the age of two years [3]. The presence of pluripotent cells within the vitelline duct remnants accounts for the development of heterotopic tissues in up to 30-50% of cases [4]. Among these, gastric mucosa is the most frequently encountered ectopic tissue, observed in 60-80% of cases, while pancreatic tissue is found in approximately 1-6% of cases. Other ectopic tissues, such as colonic, duodenal, endometrial, and biliary epithelium, have also been reported, albeit less commonly [4].

Despite its congenital nature, MD remains asymptomatic in the majority of individuals. However, approximately 1% of those with MD may experience complications, which can manifest in a variety of clinical presentations [5]. These include painless lower gastrointestinal bleeding, intestinal obstruction, inflammation (diverticulitis), and, in some cases, perforation leading to peritonitis. The relative incidence of these complications has been estimated as gastrointestinal bleeding (approximately 40%), intestinal obstruction (30%), diverticulitis (20%), and intestinal perforation (10%) [6].

In this context, we report a series of six pediatric cases with varied presentations of MD, highlighting the clinical spectrum, surgical interventions undertaken, and histopathological findings to underscore the importance of early diagnosis and timely management in symptomatic cases.

## Materials and methods

This case series examines six pediatric patients who underwent surgical intervention (laparotomy/laparoscopy/umbilical exploration) for varied presentations of MD at a tertiary care industrial hospital in Jamshedpur, over a study period from June 2022 to May 2025. IRB approval was obtained for this retrospective study from the Tata Main Hospital Institutional Ethics Committee (TMH/IEC/187/2025). The age group had a range of one month to 60 months, with an average age of 40.5 months. Only those patients were included in the study whose symptomatology was attributed to MD on final diagnosis. All patients with acute abdomen whose symptomatology was not due to MD were excluded from the study.

**Figure 1:**
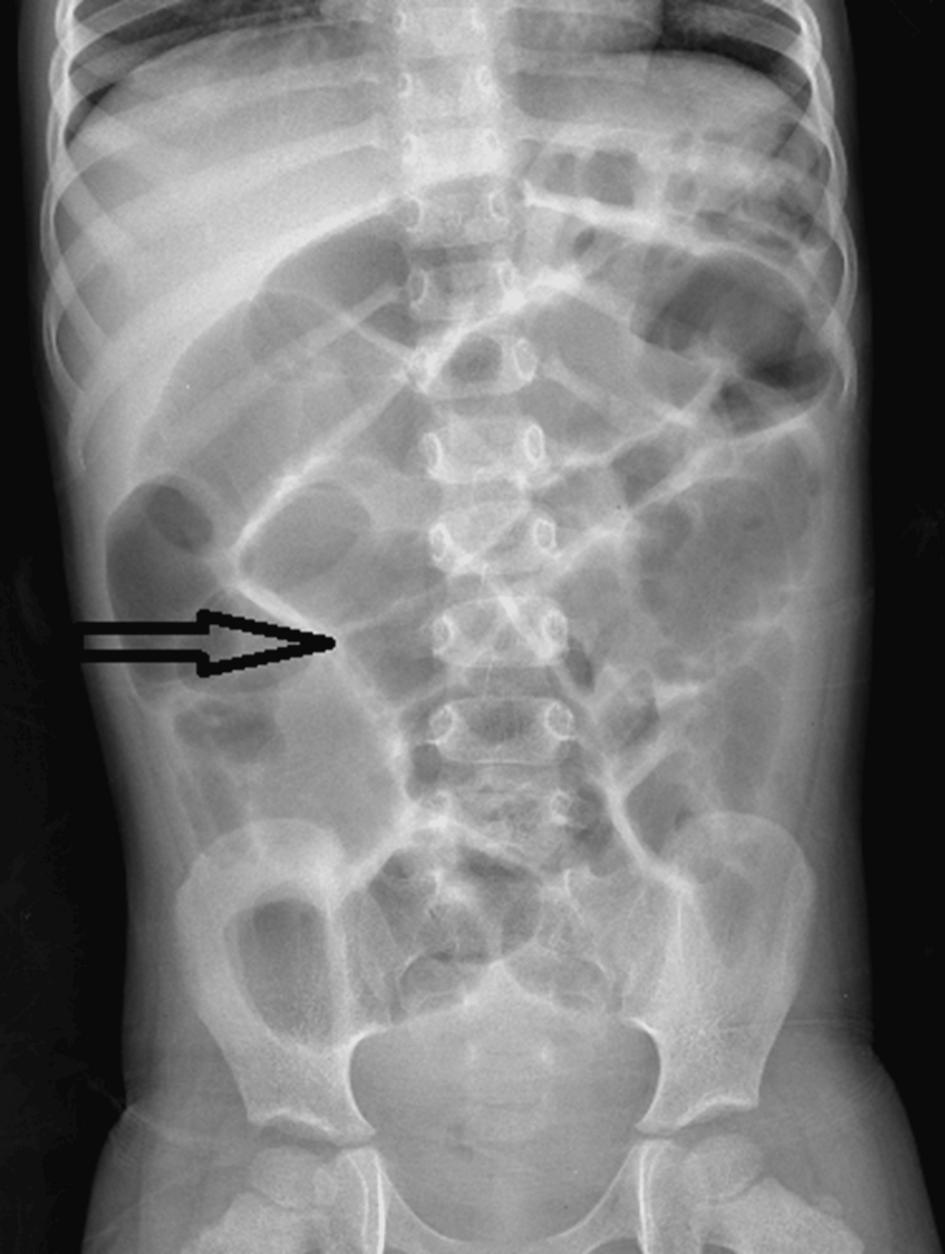
X-ray of the abdomen and pelvis showing dilated small bowel loops (arrowhead). Arrowhead showing dilated small bowel loops.

**Figure 2:**
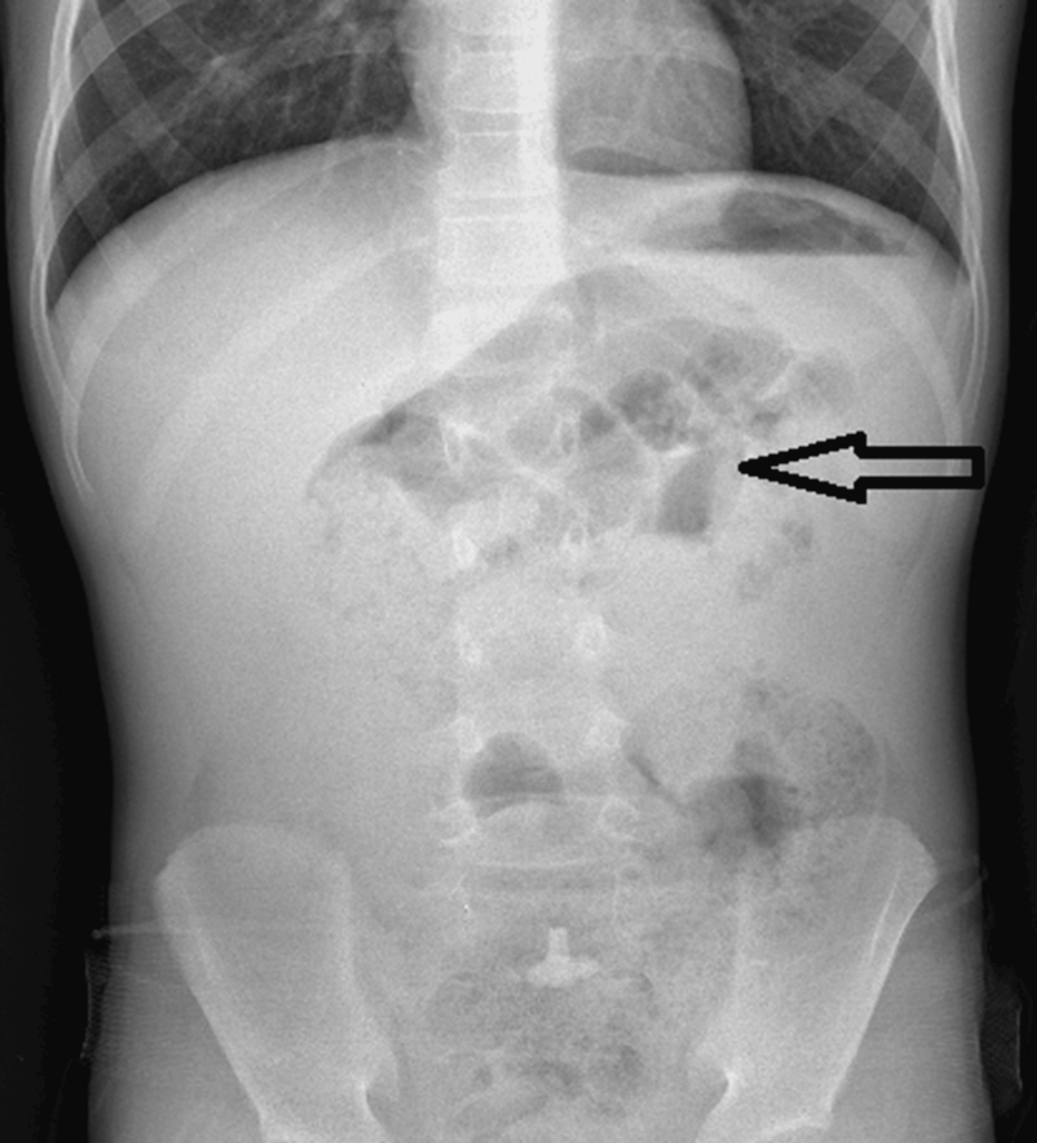
X-ray of the abdomen and pelvis showing multiple air fluid levels (arrowhead). Arrowhead showing multiple air fluid levels.

As a part of the standard protocol of any patient presenting with an acute abdomen, all patients underwent relevant blood investigations and resuscitation. The radiological investigations were limited to a skiagram of the abdomen and pelvis, the most common finding of which was multiple air fluid levels in the case of intestinal obstruction due to Meckel diverticular band (Figures 1, 2).

Ultrasonography findings were limited to the presence of dilated bowel loops in two (33%) cases presenting with intestinal obstruction. In one (16%) case, the main presentation was umbilical discharge, and the ultrasound was suggestive of subumbilical cystic swelling. The diagnostic armamentarium included a Tc-99 isotope scan (Meckel scan), which showed ectopic uptake of the radioisotope in one (16%) patient with presentation of bleeding per rectum and anemia. The blood, ultrasound, and Meckel scan findings are summarized in Table 1.

**Table 1:** Clinical presentation of Meckel's diverticulum cases. gm/dl: gram per deciliter.

S. No.	Age (months)	Signs and symptoms	Hemoglobin (g/dl)	Normal hemoglobin range (g/dl)	X-ray	Ultrasound	Meckel scan
1	1	Umbilical discharge	12.0	11–13	Normal	Sub-umbilical swelling	Not done
2	2	Umbilical discharge	8.0	11–13	Normal	Sub umbilical swelling	Not done
3	48	Abdominal pain and distension	10.0	11–13	Multiple air fluid levels	Dilated bowel loops	Not done
4	60	Abdominal pain, distension, shock	9.0	11–13	Multiple air fluid levels	Dilated bowel loops, echogenic fluid in the abdomen	Not done
5	60	Abdominal pain, distension, shock	8.5	11–13	Pneumoperitoneum	Echogenic free fluid	Not done
6	72	Anemia, intermittent abdominal pain	6.0	11–13	Normal	Normal	Ectopic isotope uptake in the right iliac fossa

**Figure 3:**
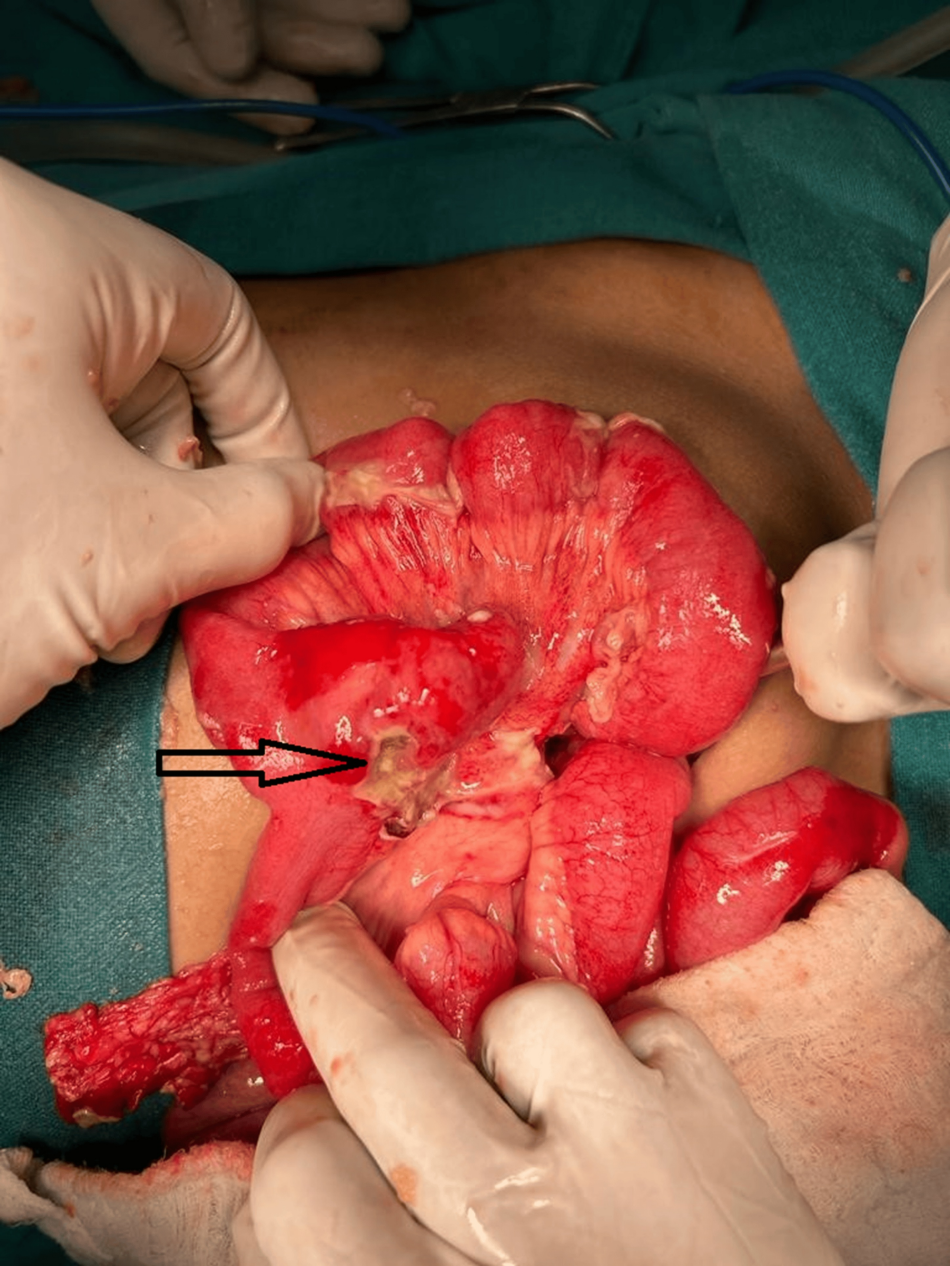
Arrowhead showing perforated Meckel's diverticulum (MD). Arrowhead showing perforated MD at base.

The mode of treatment was as per standard surgical principles, with the two (33%) patients presenting with acute intestinal obstruction undergoing resuscitation and exploratory laparotomy, followed by resection and anastomosis of the ileum. In one (16%) patient presenting with perforation peritonitis, laparotomy, segmental resection of the ileum with perforated MD (Figure 3), and double-barrel ileostomy were done as a life-saving measure due to unstable vitals, followed by ileostomy closure after eight weeks.

**Figure 4:**
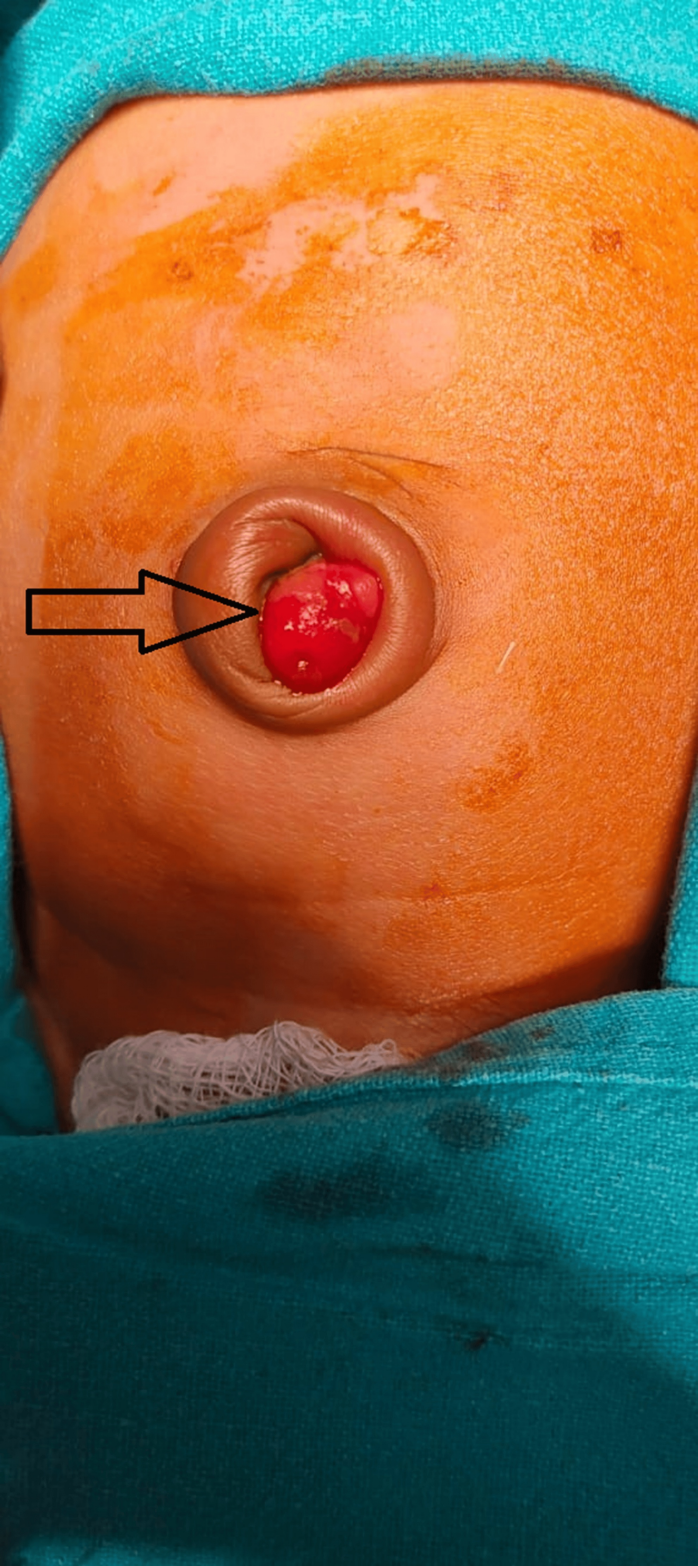
Arrowhead showing prolapsed Meckel's diverticulum (MD) through the umbilicus. Arrowhead showing prolapsed MD through the umbilicus.

**Figure 5:**
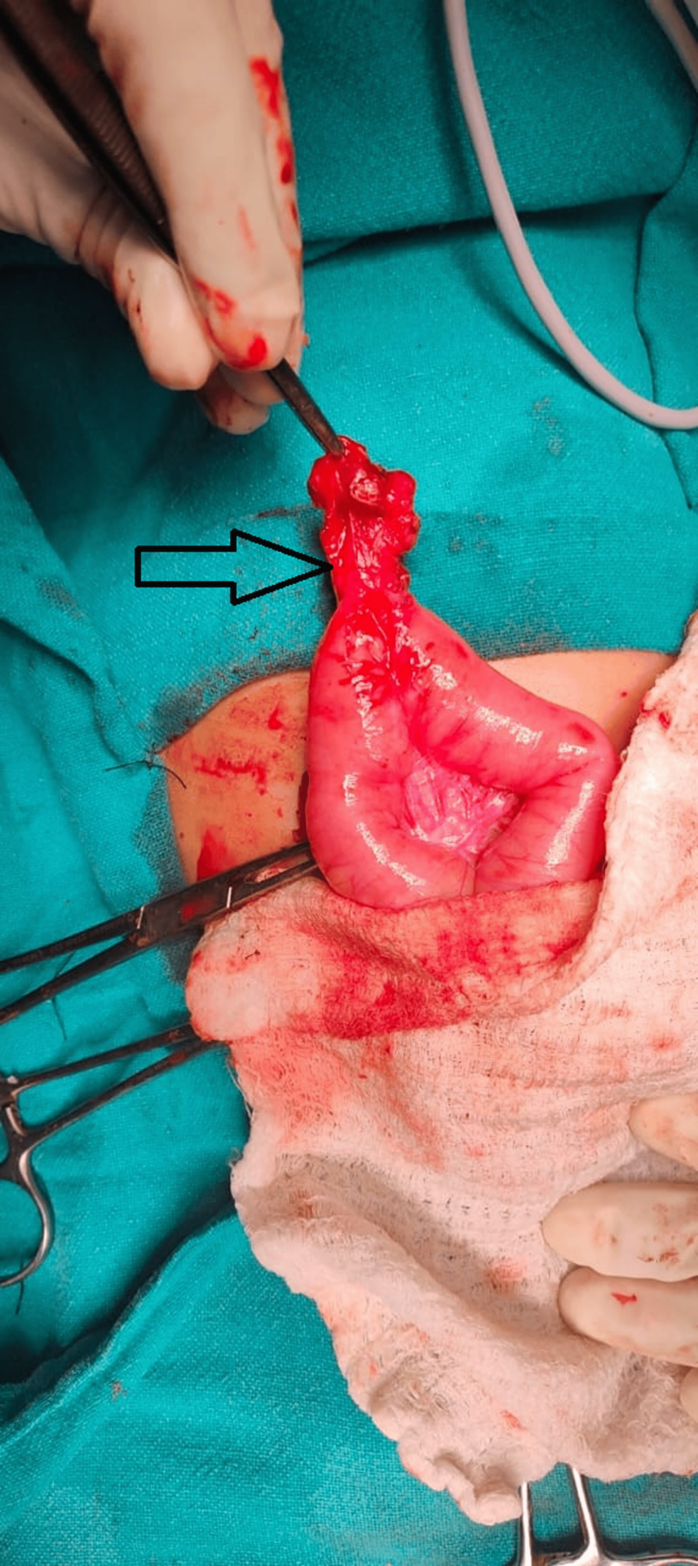
Arrowhead showing Meckel's diverticulum (MD) after umbilical exploration in the case of persistent umbilical discharge. Arrowhead showing MD after umbilical exploration in the case of persistent umbilical discharge.

The two (32%) infants who presented with umbilical discharge underwent umbilical exploration through infraumbilical smiling incision and resection anastomosis of the ileum, along with resection of MD (Figures 4, 5).

**Figure 6:**
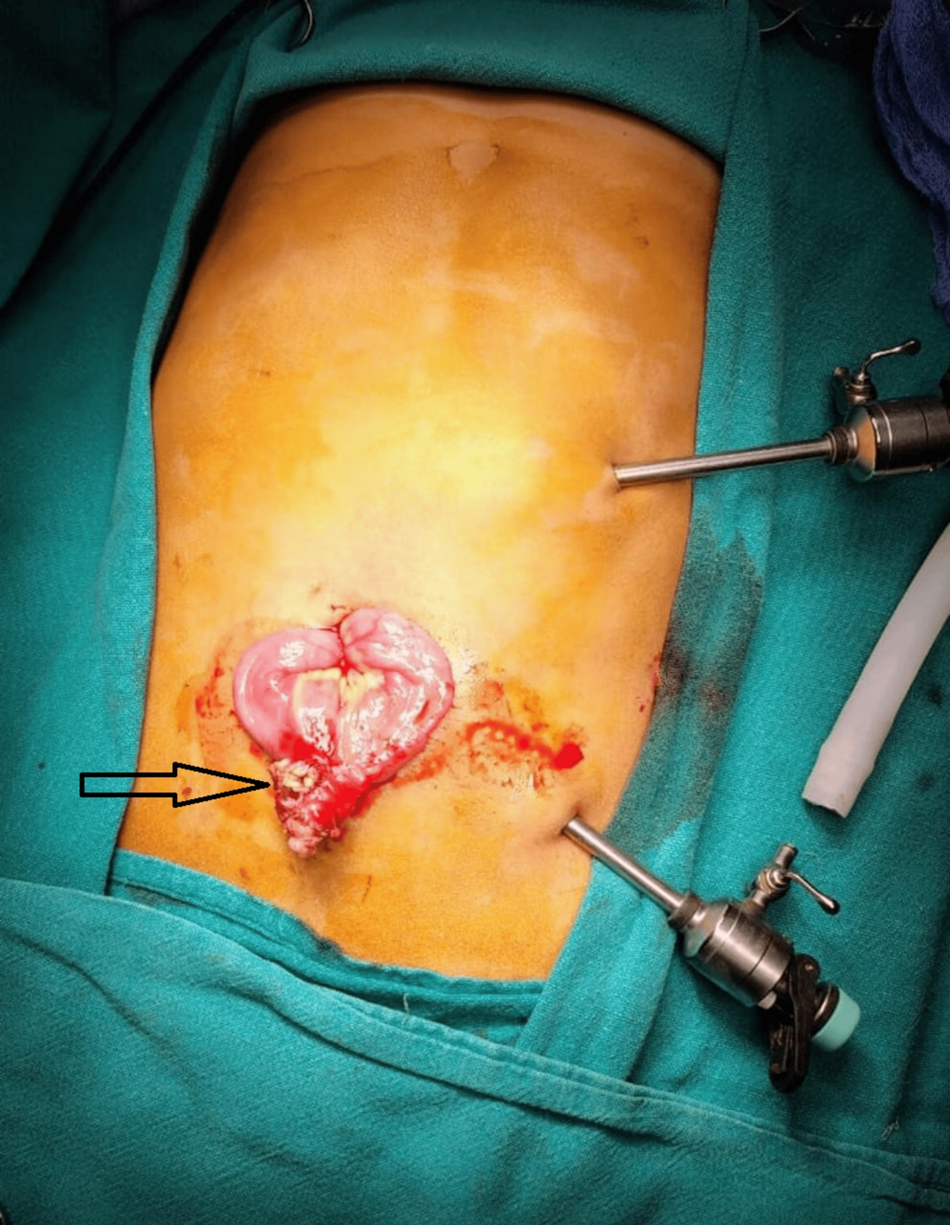
Arrowhead showing laparoscopy-assisted exteriorization of Meckel's diverticulum (MD).

One (16%) patient who had anemia and intermittent abdominal pain as presentation underwent laparoscopy-assisted Meckel diverticulectomy and extracorporeal ileo-ileal anastomosis (Figure 6).

## Results

The pediatric patients presenting with varied presentations of MD over the last three years were included in this retrospective study. The male-to-female ratio was 2:1. The mean age of presentation was 40.5 months (minimum = one month, maximum = 60 months). The most common mode of presentation was intestinal obstruction (33%), due to the Meckel diverticular band, with a mean age of 36 months at presentation. In two (33%) patients, with age at presentation of one month and two months, respectively, the main symptom at presentation was persistent umbilical discharge. There were no symptoms suggestive of intestinal obstruction or gastrointestinal bleeding in these cases. The only clinical sign suggestive was an umbilical growth and persistent umbilical discharge. Gastrointestinal bleeding with anemia was found in the 72-month-old girl, whose main symptom was recurrent abdominal pain. A Meckel scan was done, and it was suggestive of the isotope uptake in the right iliac fossa. Diagnostic laparoscopy was done, which confirmed the diagnosis of MD, and laparoscopy-assisted Meckel diverticulectomy and ileo-ileal anastomosis were done. None of the resected specimens had malignant foci on histopathological analysis. Ectopic gastric mucosa was found in five (82%) cases, but in one (16%) specimen, pancreatic acini were seen on microscopy. Post procedure, all the cases have been doing well.

## Discussion

MD represents the most prevalent congenital malformation of the gastrointestinal tract. As a true diverticulum, it comprises all three layers of the intestinal wall and frequently harbors heterotopic tissue, most commonly gastric mucosa, followed by pancreatic acinar tissue [7]. Embryologically, MD results from the incomplete obliteration of the vitelline (omphalomesenteric) duct, and the extent of this failure determines the nature and severity of clinical manifestations [7]. The presence of ectopic gastric mucosa is often the underlying cause of rectal bleeding and resultant anemia. This bleeding may present subtly, manifesting as unexplained anemia and abdominal pain in outpatient settings, or it may appear acutely as massive lower gastrointestinal hemorrhage accompanied by pallor and hypotension [8].

Another frequent clinical presentation is intestinal obstruction. This may result from a fibrous band due to partial persistence of the vitelline duct or from intussusception, where MD serves as the lead point [9,10]. Management of such complications typically involves initial stabilization of the patient, followed by surgical resection of the diverticulum. In neonates and infants, a persistent moist umbilicus with a protruding red mass may be the only sign. Such cases must be thoroughly evaluated, as they can be mistaken for umbilical granuloma or patent urachus [11]. On surgical exploration, these patients often have a cystic mass beneath the umbilicus communicating with the ileum, distinguishing MD from urachal anomalies, where the tract connects the umbilicus to the bladder.

There is ongoing debate about whether to perform a segmental ileal resection or a wedge resection when managing MD. Palpation and visual assessment during surgery are unreliable in identifying ectopic tissue. However, studies suggest that the anatomical configuration, particularly the height-to-diameter ratio, can help predict the location of ectopic mucosa. In longer diverticula (height-to-diameter ratio >2), ectopic tissue is typically located at the tip or body, whereas in shorter, broader diverticula, it can be diffusely distributed, including at the base [12]. Since hemorrhage in MD usually originates from adjacent ileal mucosa ulcerated by acid secretions from ectopic gastric mucosa, formal resection and anastomosis are often preferred to ensure the removal of both the diverticulum and the adjacent ulcerated segment [13]. In critically ill patients with intestinal gangrene and systemic sepsis, a temporary life-saving ileostomy may be necessary, with restoration of continuity through ileostomy reversal at a later date.

In this study, two (33%) out of six cases presented with intestinal obstruction due to a Meckel band. One (16%) patient arrived in hypovolemic shock with pneumoperitoneum. This child underwent emergency laparotomy, resection of the perforated MD, and creation of a double-barrel ileostomy, followed by reversal after eight weeks.

The diagnosis of MD often hinges on a high index of clinical suspicion, especially in pediatric patients presenting with anemia, abdominal pain, or rectal bleeding, once common causes like juvenile polyps or infectious colitis have been excluded. While anemia is a common hematologic finding in bleeding MD, it may not be informative in cases presenting with obstruction or umbilical discharge. Imaging studies such as abdominal ultrasound might reveal a subumbilical mass communicating with bowel loops or dilated loops in obstructive presentations. Similarly, plain abdominal radiographs may show multiple air-fluid levels, though these findings are nonspecific [14]. In cases of perforation due to acid-induced ileal ulceration, pneumoperitoneum may be detected on imaging [15]. In hemodynamically stable patients, a radionuclide scan (Meckel scan) using technetium-99m pertechnetate may aid in diagnosis by highlighting ectopic gastric mucosa in the right lower quadrant [16].

In this case series, two (33%) patients presented with umbilical discharge, one (16%) had pneumoperitoneum secondary to perforation, and one (16%) had chronic anemia with recurrent abdominal pain. The Meckel scan in this case revealed radionuclide uptake in the right iliac fossa. The patient underwent a laparoscopic-assisted diverticulectomy with extracorporeal ileo-ileal anastomosis.

There remains considerable controversy over the management of incidentally discovered MD during abdominal surgery for unrelated conditions. Traditional teaching discourages resection of a broad-based, asymptomatic MD; however, newer evidence supports prophylactic removal if the patient’s condition permits [17]. This is primarily due to the malignant potential of MD, with reports suggesting metastatic disease in 26-33% of malignancy-associated MD cases [18]. The most common malignancies reported in MD include neuroendocrine tumors, gastrointestinal stromal tumors, adenocarcinomas, metastases, and lymphomas, in descending order of frequency [19]. Nonetheless, current evidence is insufficient to justify routine prophylactic resection of MD in the absence of other surgical indications [20].

Limitations of the study

This retrospective study, although informative, is limited by several factors. Firstly, the small sample size of only six pediatric patients restricts the statistical power and generalizability of the findings. As a single-center study conducted in a tertiary care industrial hospital, the results may not reflect the broader pediatric population, especially in different geographic or healthcare settings. Secondly, due to the retrospective nature of the study, there is an inherent risk of selection bias and missing data, particularly regarding subtle clinical features and radiological details that may not have been comprehensively documented in patient records. The diagnostic approach was also constrained by the limited availability of advanced imaging modalities; Meckel scans, for instance, were performed in only one case, potentially affecting the accuracy of preoperative diagnosis in the rest. Furthermore, long-term follow-up outcomes such as postoperative complications, growth, and nutritional status were not evaluated in detail. Lastly, the decision-making regarding surgical approach (laparotomy vs. laparoscopy) and the extent of resection was based on individual surgeon discretion, which could introduce variability in management and outcomes.

## Conclusions

MD, a true diverticulum, can remain clinically silent or may get symptomatic with life-threatening complications like massive bleeding per rectum, intestinal obstruction, or perforation peritonitis. The treatment is usually straightforward in the form of abdominal exploration and resection of MD, followed by ileo-ileal anastomosis or ileostomy in sick cases. In clinically indolent cases presenting with umbilical discharge or persistent anemia due to occult bleeding per rectum, it requires a high index of suspicion and specific investigations like Tc-99 radioisotope scan to clinch the diagnosis, though in most cases, MD is an incidental intra-abdominal finding. Incidentally detected MD during abdominal exploration for another cause should be resected due to their malignant and metastatic potential, though available literature does not justify prophylactic removal of MD without any other indication for abdominal surgery.
